# Investigation of oxidative stress in pterygium tissue

**Published:** 2011-02-09

**Authors:** Mehmet Balci, Şemsettin Şahin, Fatih Mehmet Mutlu, Ramazan Yağci, Pınar Karanci, Metin Yildiz

**Affiliations:** 1 Department of Ophthalmology, Abdurrahman Yurtaslan Oncological Training and Research Hospital, Ankara, Turkey; 2 Gaziosmanpaşa University Medical School, Department of Biochemistry, Tokat, Turkey; 3 Gülhane Academy of Military Medicine, Department of Ophthalmology, Ankara, Turkey; 4 Fatih University, Medical School, Department of Ophthalmology, Ankara, Turkey

## Abstract

**Purpose:**

To investigate changes in the oxidant-antioxidant system in pterygium tissue.

**Methods:**

Tissue samples ablated from 40 patients during pterygium surgery constituted the study material, while normal nasal conjunctiva tissue samples from 20 patients matched for age group (who had undergone surgery for strabismus or extracapsular cataract surgery) were used as controls. The samples were kept at −70 °C until the time of analysis. Catalase (CAT), superoxide dismutase (SOD), and glutathione peroxidase (GSH-Px) enzymatic activity and the levels of nitric oxide (NO) and malone dialdehyde (MDA) were studied in both groups.

**Results:**

The mean age was 54.0±12 years in the pterygium patients and 49.0±19 in the controls (p=0.270). The enzyme activity levels were significantly lower in the pterygium group when compared to the controls (p<0.001 in each case), while in the same group a significant increase was observed in the MDA and NO levels (also p<0.001, in both cases).

**Conclusions:**

These findings indicating oxidative stress in the pterygium tissue suggest that oxidative stress can play a role in pterygium etiopathogenesis.

## Introduction

Pterygium is an epithelial hyperplasia characterized by the presence of fibro-vascular tissue, thought to be formed by the advancement of transformed conjunctival tissue into the cornea [1]. A role of ultraviolet (UV) radiation damage, irritation, or inflammation is hypothesized in its etiopathogenesis; the form, incidence, and distribution of pterygium support the possible role of UV radiation [2,3]. The peripheral focus theory [4,5] and the acute and chronic tissular inflammation that has been demonstrated to be an effect of UV radiation also support the thesis that such radiation is an important factor in the pathogenesis of pterygium [6-8].

UV radiation acts directly by phototoxicity or indirectly through free radicals (FR.) Intracellularly, the FR cause oxidative damage by acting on macromolecules such as proteins, lipids, and nucleic acids. The FR, which consist principally of molecules like the superoxide anion (O_2_^-^), hydrogen peroxide (H_2_O_2_) and peroxynitrite (ONOO^-^), are detoxified by enzymes such as superoxide dismutase (SOD), catalase (CAT), and glutathione peroxidase (GSH-Px.) The antioxidant system and the amount of FR are kept in a certain state of homeostasis. When exogenous or endogenous factors increase oxidative stress, homeostasis is disturbed and the FR denature many basic intracellular molecules like nucleic acids, proteins, and lipids. As for malone dialdehyde (MDA), a product of the breakdown into their essential chains of mainly unsaturated fatty acids through the oxidation mechanism, MDA is accepted as a reliable marker of the lipid peroxidation that occurs as a result of oxidative stress.

It has been reported in recent times that certain growth factors, such as the vascular endothelial growth factor (VEGF), and cellular mediators like nitric oxide (NO), also play a role in the pathogenesis of pterygium [9,10]. It is also stated that NO, primarily secreted by the endothelium and hypothesized to play a role in vasodilation, neurotransmission, and inflammation, also takes part in vascular tonus, vascular permeability, and capillary vessel growth [9]. It has been observed to act as a protective antioxidant agent at physiologic doses, and when administered in large amounts it has oxidating and apoptotic properties and enhances VEGF production [11,12]. It is also known that UV radiation increases NO release also by non-enzymatic means [13]. All these observations concur to suggest that vasoproliferative processes and their contributing factors play a role in pterygium pathogenesis.

The presence of oxidative stress and of supporting data relative to NO does not mean that their role in the genesis of pterygium is clear yet [14,15]. The objective of our study is to expose observations on oxidative stress and changes in NO in both pterygium and the normal conjunctiva and investigate the role they play in the development of pterygium.

## Methods

Between January 2005 and March 2006, subjects who underwent surgery for pterygium (Group I), and control subjects (Group II) in matching age groups who had been operated on for strabismus or cataracts and in whom conjunctiva samples were collected from the nasal limbus area, were included in the study. Patients who had already undergone eye surgery; those afflicted with ocular diseases such as glaucoma, uveitis, and dry eye syndrome; those with systemic disease such as diabetes or rheumatoid arthritis; those with chronic addictions including those to smoking and alcohol; and those with pterygium of the atrophic type were all excluded from the study. All tissue samples were collected with the patient's consent and the approval of the Hospital Ethical Committee. Pterygium surgery was performed by dissecting the pterygium head away from the cornea and excising it en bloc with the body. During the procedure, the conjunctiva was dissected and separated as well as possible from the subconjunctival fibrovascular tissue, aiming to thus avoid conjunctival changes initiated by the presence of subconjunctival tissues. All conjunctiva samples were kept at −70 °C until the performance of the biochemical investigations. The MDA and NO levels and the activity of SOD, CAT, and GSH-Px in the samples were then measured.

MDA levels were measured by using the reaction between MDA and Thiobutyric Acid-Reactive Substance (TBARS) at 90–95 °C, which produces a pink chromogen, followed 15 min later by the spectrophotometric reading at 532 nm of the absorbance in the samples submitted to accelerated cooling [16].

The determination of NO levels were essentially performed by the diazotization N-naphthylethylene diamine (NNDA) dependent on nitrite and sulfanilamide produced by the Griess reaction and the modified cadmium reaction, and spectrophotometrically measuring the color produced by this reaction [17] at 545 nm.

The measurement of SOD activity is based on the reduction of nitroblue tetrazolium (NBT) by superoxide produced with the xanthine/xanthine oxidase system, following a modified version of the methods used by Sun et al. [18] and Durak et al. [19]. The absorbance of the resulting formazone was measured at 560 nm to determine the enzymatic activity.

GSH-Px activity was measured following the method communicated by Paglia et al. [20]. In the presence of hydrogen peroxide, GSH-Px catalyzes the oxidation of reduced glutathione to oxidized glutathione. The reduction in absorbance during the oxidation of the nicotinamide adenine dinucleotide phosphate (NADP) used in the reaction to NADP^+^ is measured to determine the enzymatic activity. CAT activity was determined by the Aebi method [21], by measuring at 240 nm the reduction in absorbance created by the decomposition (by CAT) of the added hydrogen peroxide into water and oxygen. The protein level of the supernatant was determined by Lowry's method [22]; MDA and NO levels were expressed as nmol/g protein, SOD activity as U/mg protein, CAT activity as k/g protein, and that of GSH-Px as U/g protein [21].

The software package SPSS 12.0 (SPSS for Windows, version 12.0, SPSS Inc., Chicago, IL) was used for all statistical analysis. Student's *t*-test was used to analyze continuous variables and the χ^2^ test was used to analyze categorical variables of the groups. A p-value of less than 0.05 was considered statistically significant.

## Results

The demographic characteristics of the groups and the measured enzymatic activity, and MDA and NO levels are shown in Table 1 and Table 2, respectively. There was no significant difference among the two groups as to age or sex distribution (Table 1). An important and statistically significant reduction in the enzymatic activity levels of SOD, CAT, and GSH-Px was observed in the study group as compared to those of the control group (p<0.001 for all three values.) The NO and MDA values in the study group were significantly higher than in the control group (p<0.001 for both).

**Table 1:** Demographic properties of the study population.

**Demographic properties**	**Pterygium**	**Control**	**p**
Age	54±12.0	48±19.46	0.270¶
**Sex**			0.232§
Female	26	16	
Male	14	4	
**Laterality**			0.273§
Right eye	18	11	
Left eye	22	9	

Values are given as mean±SD (¶: Student *t*-test; §: χ^2^).

**Table 2:** SOD, CAT, GSH-Px activities and MDA, NO levels.

**Enzymes**	**Pterygium**	**Control**	**p**
SOD (U/mg protein)	0.0210±0.0051	0.0446±0.0073	0.000¶
CAT (k/g protein)	0.0890±0.0328	0.4382±0.0996	0.000¶
GSH-Px (U/g protein)	0.9255±0.2038	2.8360±0.4797	0.000¶
MDA (nmol/g protein)	12.2078±1.7552	7.7482±0.2681	0.000¶
NO (μmol/g protein)	0.7586±0.0538	0.0353±0.0037	0.000¶

Values are given as mean±SD (¶: Student *t*-test; SOD: Superoxide dismutase; CAT: Catalase; GSH-Px: Glutathione peroxidase; MDA: Malondialdehyde; NO: Nitric oxide.)

## Discussion

The etiopathogenesis of pterygium is controversial. Even though there are many publications showing the influence of UV radiation [23-25], the role of the antioxidant system is still subject to discussion. We have determined in our study that, alongside the significant reduction in the enzymatic activity, there was a significant increase in the NO level. We think that these findings show the presence of oxidative stress in the etiology of pterygium and can contribute to earlier reports relative to NO [14,15].

It has been shown that the expression of NO, specifically that released by the endothelium and neuronal sources, can also be non-specifically activated by endotoxins and cytokines, and as a result that NO can be released in many different tissues [26,27]. Free oxygen radicals, diverse growth factors and cytokines, like cyto-oxygenase 2 (COX2), play a known role in skin cancers developing under the influence of UV radiation [28-32]. COX2 is a cytokine that plays a notable part in angiogenesis induced by inflammatory cytokines [33]. Chiang et al. [34] have shown the presence of COX2 in pterygial tissue and reported that it might play a role in the pathogenesis of pterygium. Studies in cornea and pterygium tissue samples have shown increases in cytokine and growth factor levels [23]. An increase in NO due to higher cytokine activity caused by UV radiation seems a possible mechanism; the increased activity of inducible nitric oxide synthase (iNOS) found in pterygium tissue also shows the presence of an enzymatic infrastructure for the NO increase [15]. Aside from all this, we should not forget that UV radiation can directly increase NO expression, by non-enzymatic means [13].

The NO increase that we found agrees with findings byother authors, especially Lee et al. [15]. The increase in serum and vitreous humor NO levels observed in diabetes and age-related macular degeneration, i.e., diseases dominated by vascular pathology and degeneration, also supports our findings [35,36]. It seems possible for high levels of NO to be a factor increasing oxidative stress by means of their oxidating and apoptotic influence [11,12,37]. Beside these influences, there are also studies showing that NO participates in angiogenesis. It was observed that angiogenesis induced by VEGF can be slowed in vivo by NO synthetase inhibition [38]. Papapetropoulos et al. [11] reported that NO participates in the angiogenic activity of VEGF. The observation by Marcovici et al. [10] of excessive quantities of VEGF in the pterygium tissue also indicates the presence of angiogenesis in the development of pterygium. Considering also that prostaglandin E_1_ (PGE_1_) and substance-P (SP) also contribute to angiogenic response [38,39], there are many factors which could contribute to the increase in NO level in pterygium. While supporting the findings by Lee et al. [15] (increased iNOS), our study results are different than those reported by Özdemir et al. [14]. In contradiction to thesis that a low NO level could be related to the fact that NO is depleted by use as a protective agent against oxidative stress, and that it also could be a result of the use of the entire pterygial tissue instead of epithelium use only, Solomon [40] proposed that this fact might be the result of hyper‐irrigation of the blood by the rich vascular net of the pterygium. This is the opposite of the ischemic conditions [35,36] through which NO levels rise. The results obtained by Özdemir et al. [14] could also have been due to clinical variables, like the condition, atrophic or not, of the pterygia that had undergone surgery. We selected cases of non-atrophic, vascularized and inflammatory pterygium of the progressive type. The active angiogenesis in such cases could be one cause for our findings of high levels of NO. Information on the clinical appearance of the pterygium cases is not found in the report by Özdemir et al. [14]. Additionally, in our samples, we dissected away the subepithelial parts developing from the Tenon capsule to as great an extent possible, to keep them as similar as possible to the control samples. Our aim in this was to avoid contamination of our results by subconjunctival tissues subject to abnormal fibro-elastotic transformation. However, such a hypothesis remains to be tested, and a comparison of levels of VEGF and NO in pterygium samples that include or lack subconjunctival tissue must still be performed in advanced studies. One other possible reason for finding high NO levels in pterygium cases could be that NO is produced in excess as a protector against the oxidative stress related to UV radiation or chronic inflammation.

We observed other powerful indications, in our study, of the presence of oxidative stress in pterygium tissue. The oxidative stress showed itself in the increased level of MDA and the reduced activity of the enzymes SOD, CAT, and GSH-Px.

The fall in antioxidant enzyme activities could be correlated to the detoxification of the possibly increased number of oxidative stress molecules. It has been shown in many degenerative processes that changes in antioxidant enzyme activities accompany an increase in lipid peroxidation [41-43]. Also, in other experimental works, UV damage has been observed to cause an increase in the MDA level in the eye [44,45] and in other tissues [46], in correlation with a fall in antioxidant enzymatic activity and an increase in lipid peroxidation. The presence of oxidative stress in pterygium has been proved by finding an increase in 8-OHdG (8-hydroxydeoxyguanosine) [24]. The significant increase (p<0.001) in MDA, consistently accompanying significant decreases (p<0.001) in the activity of SOD, CAT, and GSH-Px in our study, leads us to think that oxidative stress is an important mechanism in the development of pterygium.

In this study, we evaluated NO, the antioxidant system enzymes, and lipid peroxidation together for the first time. Our results show that oxidative stress plays a role in the etiopathogenesis of pterygium. We cannot forget, however, that as much as UV radiation is an important factor in the pathogenesis of oxidative stress, chronic inflammation also plays a part in the development of this lesion, and oxidative stress can increase with inflammation. To conclude, oxidative stress and NO do play a role in the development of pterygium, and this complex process must be kept in mind in the prevention and treatment of pterygium.
